# Association between the Japanese Diet and Coronary Artery Disease in Patients Undergoing Coronary Angiography

**DOI:** 10.3390/nu15102406

**Published:** 2023-05-21

**Authors:** Yukihiko Momiyama, Yoshimi Kishimoto, Emi Saita, Masayuki Aoyama, Reiko Ohmori, Kazuo Kondo

**Affiliations:** 1 Department of Cardiology, National Hospital Organization Tokyo Medical Center, 2-5-1 Higashigaoka, Tokyo 152-8902, Japan; 2 Department of Food Science and Human Nutrition, Faculty of Agriculture, Setsunan University, Osaka 573-0101, Japan; 3 Research Institute of Environmental Medicine, Nagoya University, Nagoya 464-8601, Japan; 4 Department of Cardiovascular Medicine, Toho University Graduate School of Medicine, Tokyo 143-8540, Japan; 5 Faculty of Regional Design, Utsunomiya University, Tochigi 321-8505, Japan; 6 Ochanomizu University, Tokyo 112-8610, Japan

**Keywords:** coronary angiography, coronary artery disease, food frequency questionnaire, Japanese diet, myocardial infarction

## Abstract

Several cohort studies have reported that the Japanese diet is associated with reduced cardiovascular disease mortality. However, the results were not always consistent, and most of those studies conducted dietary surveys around 1990. We investigated the association between the Japanese diet and coronary artery disease (CAD) in 802 patients undergoing coronary angiography. The Japanese diet score was defined as the sum of scores of the intakes of fish, soy products, vegetables, seaweed, fruits, and green tea. CAD was found in 511 patients, of whom 173 had myocardial infarction (MI). Intakes of fish, soy products, vegetables, seaweed, fruits, and green tea were lower in patients with CAD, especially in those with MI, than in those without CAD. As a result, the Japanese diet score was significantly lower in patients with CAD than in those without CAD (*p* < 0.001). To clarify the association between the Japanese diet and CAD, the 802 study patients were divided into three tertiles by the Japanese diet score. The proportion of CAD decreased with the Japanese diet score, reaching 72% in patients at T1 (lowest score), 63% at T2, and 55% at T3 (highest) (*p* < 0.05). The proportion of MI also decreased with the Japanese diet score, reaching 25% at T1, 24% at T2, and 15% at T3 (*p* < 0.05). In a multivariate analysis, compared with T1, the adjusted odds ratios for CAD and MI were 0.41 (95% confidence interval [CI]: 0.26–0.63) and 0.61 (95% CI: 0.38–0.99) for T3, respectively. Thus, the Japanese diet was found to be inversely associated with CAD in Japanese patients undergoing coronary angiography.

## 1. Introduction

Japanese people are known to have the longest life expectancy in the world mainly due to a very low mortality rate from coronary artery disease (CAD), considered to be attributable to their high intakes of fish; plant-based foods, such as soy products; and green tea—making up the so-called Japanese diet [1,2,3]. Suzuki et al. [4] reviewed the characteristics of the Japanese diet from epidemiological studies focusing on diet. The authors found that soy products, fish, vegetables, rice, miso soup, seaweed, pickles, fruits, and green tea were the characteristic components of the Japanese diet. Because of its possible association with a low incidence and mortality of CAD in Japanese people, the Japanese diet has attracted considerable attention since the 1960s [5]. Recently, the Japan Atherosclerosis Society also recommended the adoption of an increased intake of fish, soy products, vegetables, seaweed, fruits, and unrefined grains as “The Japan Diet” for the prevention of atherosclerotic diseases [6].

In Japan, one cohort study reported that adherence to the Japanese diet was associated with reduced mortality from heart disease (HD), including CAD [7], whereas another study failed to show a significant association between the Japanese diet and CAD mortality [5]. A recent meta-analysis of four cohort studies in the Japanese population showed that higher adherence to the Japanese diet was associated with lower HD/CAD combined mortality [8]. However, most of those cohort studies conducted their dietary surveys around 1990, and the dietary pattern in the Japanese population changed with a trend toward decreasing intakes of rice, fish, and soy products [8,9]. Furthermore, the changes in individual dietary habits during >10 years of follow-up were not considered [7]. In contrast, several cross-sectional studies in patients undergoing coronary angiography (CAG) reported an association between CAD and dietary patterns when CAD was diagnosed. One study in Germany reported that adherence to the Mediterranean diet was inversely associated with the presence of severe CAD in patients undergoing CAG [10]. In Greece, the Western diet was shown to be positively associated with severe CAD in patients with CAD [11]. However, in Japan, studies showing the associations between the intakes of Japanese foods or beverages and CAD in patients undergoing CAG are scarce. In Japanese patients undergoing CAG, two studies [12,13] reported that green tea intake was inversely associated with the presence of CAD. One case–control study reported that tofu intake was significantly associated with myocardial infarction (MI) only in women [14]. However, no study has reported the association between the Japanese diet and CAD or MI in patients undergoing CAG. We recently reported that the intake of green tea, but not that of coffee, was inversely associated with the presence of CAD and MI in 612 Japanese patients undergoing CAG [15]. Therefore, the present study extends our previous report by increasing the number of study patients and elucidating the association between the Japanese diet and the presence and severity of CAD in 802 patients undergoing CAG.

## 2. Materials and Methods

### 2.1. Study Patients

**Figure 1:**
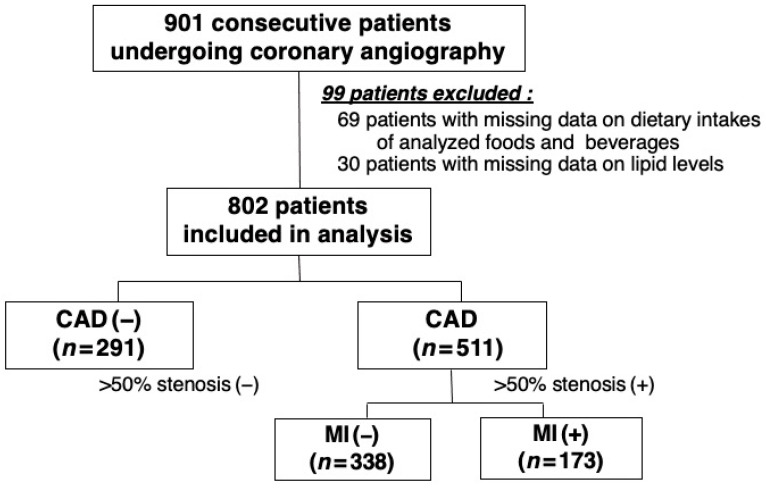
Patient selection flow chart.

In 2008, we started our study to evaluate the dietary intake as well as the clinical and angiographic data of patients undergoing CAG at the NHO Tokyo Medical Center. Our study was a hospital-based, observational, cross-sectional study and was approved by the institutional ethics committee of our hospital (Approval No. R08-050/R21-037). In line with the Declaration of Helsinki, all study patients gave their written informed consent prior to their participation. However, patients with a history of percutaneous coronary intervention or cardiac surgery or those undergoing hemodialysis were not asked to participate in our study. A total of 901 consecutive patients who underwent CAG for the first time for suspected CAD were enrolled. In the present study, the dietary intake was evaluated using a food frequency questionnaire (FFQ) and then compared with clinical and angiographic data. Of the 901 patients, 69 with missing data on the dietary intake of analyzed foods and beverages and 30 with missing data on lipid levels were excluded. As a result, 802 patients were included and analyzed in the present study (Figure 1).

Blood samples were taken in a fasting state before CAG, and serum lipid levels were measured by standard laboratory methods. However, in patients undergoing emergent CAG, such as patients with MI, blood samples were taken within a few days after admission. Hypertension was defined as a blood pressure of ≥140/90 mmHg and/or the use of anti-hypertensive drugs; 442 (55%) patients were taking anti-hypertensive drugs. Hypercholesterolemia was defined as an LDL-cholesterol level of >140 mg/dL and/or the use of statins; 279 (35%) patients were taking statins. Diabetes mellitus (DM) was defined as a fasting plasma glucose level of ≥126 mg/dL and/or being treated for DM, and 215 (27%) patients were diabetic.

### 2.2. Assessments of Coronary Angiograms

CAG was conducted using a cine angiogram system (Philips Electronics Japan, Tokyo, Japan). CAD was defined as at least one coronary artery with >50% luminal diameter stenosis on angiograms. MI was confirmed by the documentation of coronary artery stenosis plus either elevated cardiac enzymes and/or diagnostic electrocardiographic changes. The severity of CAD was assessed as the number of >50% stenotic coronary vessels and those of >50% and >25% stenotic segments. Coronary artery segments were divided into 29 segments by the Coronary Artery Surgery Study classification. All angiograms were assessed by a single cardiologist who was blinded to the patients’ clinical and dietary data.

### 2.3. Assessments of the Dietary Intake

We assessed the intakes of foods and beverages using a semiquantitative FFQ filled out by the study patients. The intakes of fish, soy products, green/yellow vegetables, seaweed, and fruits as well as meat were determined and classified into 3 categories (<3 times/week, 3–4 times/week, and >4 times/week). Regarding beverages, the intakes of green tea and coffee were determined by 3 categories (<1 cup/day, 1–3 cups/day, and >3 cups/day), as was the intake of alcohol (<1 time/week, ≥1 time/week, and every day). As shown in our recent report [15], the FFQ used in the present study was modified from that used in our earlier studies performed at the National Defense Medical College Hospital [16,17], particularly with regard to collecting data on the intakes of fish and seaweed.

The Japanese diet is reported to be characterized by high intakes of fish, soy products, vegetables, white rice, miso soup, seaweed, pickles, fruits, and green tea [4]. However, because most Japanese people eat rice as a staple food almost every day and because no significant association between the rice intake and cardiovascular disease (CVD) was reported in a Japanese population [18] and in a US population [19], we did not evaluate the rice intake. Furthermore, miso soup was not taken into account in the intake of soy products, as the amount of soy protein and isoflavone in miso soup is much less than in tofu and other dishes [14]. Given that a cohort study reported that the Japanese diet was highly correlated with high intakes of fish, soy products, vegetables, seaweed, fruits, and green tea [5], we also defined the Japanese diet score as the sum of the scores of the intakes of five foods (fish, soy products, vegetables, seaweed, and fruits) and one beverage (green tea). The intake of each of these five foods as well as green tea was scored from 0 to 2 points, so the Japanese diet score ranged from 0 to 12.

### 2.4. Statistical Analyses

We conducted all statistical analyses using the SPSS software package, ver. 25 (IBM, Tokyo, Japan) and a *p* value of <0.05 was considered to indicate statistical significance. Continuous and categorical variables were expressed as the mean and standard deviation (SD) and the number and percentage (%), respectively. For continuous variables, an unpaired *t*-test was used to evaluate differences between two groups, while an analysis of variance with the Bonferroni correction was used for multiple comparisons. For categorical variables, chi-square tests were used for differences between two groups, while cross-tabulations with the Bonferroni correction were used for multiple comparisons. Nonparametric variables, such as the numbers of stenotic coronary segments, were expressed as the median and interquartile range. For nonparametric variables, the Mann–Whitney U test was used for differences between two groups, while the Kruskal–Wallis test followed by the Dunn test with the Bonferroni correction was used for multiple comparisons. The correlations between the Japanese diet score and the severity of CAD were assessed by Spearman’s rank correlation test. Furthermore, we performed a receiver–operating characteristic curve analysis to determine the optimal cut-off value of the Japanese diet score for CAD and found that the optimal cut-off value was 6, as this showed the highest Youden index.

To clarify the association between the Japanese diet and CAD, the 802 study patients were divided into 3 tertiles according to the Japanese diet score: first tertile (T1), score 0–4; second tertile (T2), score 5–7; and third tertile (T3), score 8–12. The T1 group was used as the reference group, which corresponds to that with the lowest intake of the Japanese diet. Furthermore, we performed a multiple logistic regression analysis to elucidate the independent associations between the Japanese diet and the presence of CAD, three-vessel disease (3-VD), or MI. In this analysis, the dependent variables were the presence of CAD, 3-VD, or MI. The adjusted covariates were as follows: (1) age and sex (model 1); (2) age, sex, hypertension, hypercholesterolemia, HDL-cholesterol levels, statin use, DM, and smoking (model 2); and (3) the covariates in model 2 plus the intake of meat, coffee, and alcohol (model 3).

## 3. Results

**Figure 2:**
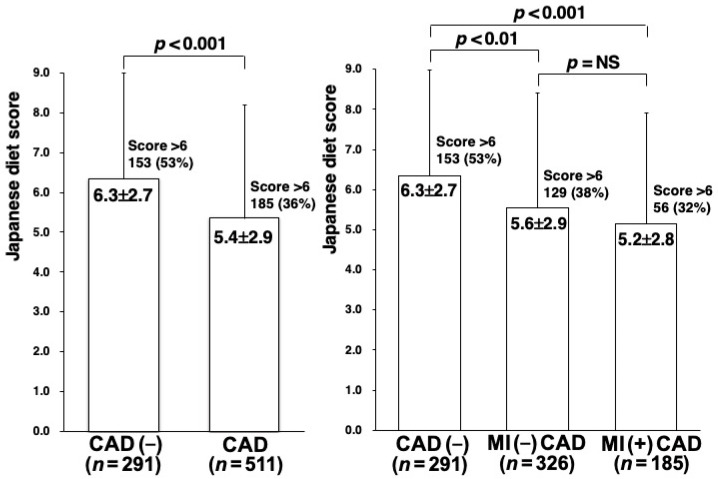
Japanese diet scores in patients with and without CAD. The Japanese diet score was significantly lower in patients with CAD than in those without CAD (left). Furthermore, it tended to be lower in CAD patients with MI than in those without MI, but this difference did not reach statistical significance (right). In addition, the proportion of patients with a score >6 was significantly lower in patients with CAD (36%), especially those with MI (32%), than in those without CAD (53%).

Among the 802 study patients, CAD was angiographically found in 511 (64%), of whom 148 (18%) and 173 (22%) were found to have 3-VD and MI, respectively. Compared with the 291 patients without CAD, the 511 patients with CAD were significantly older and had a male predominance; a higher proportion of hypertension, DM, smoking, and hypercholesterolemia; and lower HDL-cholesterol levels. However, compared with the 338 CAD patients without MI, the 173 CAD patients with MI were younger and had a lower proportion of hypertension and DM, higher LDL-cholesterol levels, and lower HDL-cholesterol levels (Table 1). Regarding the intake of foods and beverages, the intakes of fish, soy products, vegetables, seaweed, and fruits as well as green tea were significantly lower in patients with CAD, especially those with MI, than in those without CAD (Table 2). As a result, the Japanese diet score was found to be significantly lower in patients with CAD (5.4 ± 2.9) than in those without CAD (6.3 ± 2.7) (*p* < 0.001). Furthermore, the Japanese diet score tended to be lower in CAD patients with MI (5.2 ± 2.8) than in those without MI (5.6 ± 2.9), but this difference did not reach statistical significance (Table 2) (Figure 2). In addition, the proportion of patients with a Japanese diet score of >6 was significantly lower in patients with CAD (36%), especially in those with MI (32%), than in those without CAD (53%) (*p* < 0.001) (Table 2), indicating that the sensitivity and specificity of a Japanese diet score >6 for CAD (−) were 53% and 64%, respectively.

To clarify the association between the Japanese diet and CAD, the 802 study patients were divided into three groups according to the Japanese diet score. The proportion of CAD decreased in a stepwise manner with the Japanese diet score, reaching 72% in patients in the T1 group, 63% in the T2 group, and 55% in the T3 group (*p* < 0.001). The number of >25% stenotic coronary segments also decreased with the Japanese diet score, reaching 3.0 (median) in the T1 group, 2.0 in the T2 group, and 1.0 in the T3 group (*p* < 0.001) (Table 3). Furthermore, the numbers of >50% and >25% stenotic segments were found to be significantly and inversely correlated with the Japanese diet score (Spearman’s rank correlation test: r = −0.12 and r = −0.14, respectively, *p* < 0.002). The proportion of MI also decreased in a stepwise manner with the Japanese diet score, reaching 25% in patients in the T1 group, 24% in the T2 group, and 15% in the T3 group (*p* < 0.01) (Table 3).

Table 4 summarizes the data regarding the associations between the Japanese diet score and the presence of CAD, 3-VD, and MI obtained by multiple logistic regression analyses. The proportion of CAD, 3-VD, and MI significantly decreased with the Japanese diet score, even after adjusting for age, sex, atherosclerotic risk factors, and the intake of meat, alcohol, and coffee. Compared with the T1 group, which had the lowest Japanese diet score of 0–4, the adjusted odds ratios for CAD, 3-VD, and MI were 0.41 (95% confidence interval [CI]: 0.26–0.63), 0.45 (95% CI: 0.27–0.74), and 0.61 (95% CI: 0.38–0.99) for the T3 group, which had the highest score of 8–12, respectively. However, as shown in Table 4, the final models (model 3) showed poor overall model performance for predicting CAD, 3-VD, and MI (Nagelkerke R^2^ values = 0.27, 0.15 and 0.18).

## 4. Discussion

In the present study, the intakes of fish, soy products, vegetables, seaweed, fruits, and green tea were lower in patients with CAD than in those without CAD, and the Japanese diet score was found to be low in patients with CAD. The proportion of CAD and MI significantly decreased with the Japanese diet score, and the severity of CAD, defined based on the number of stenotic segments, was inversely correlated with the Japanese diet score. The Japanese diet was thus found to be inversely associated with the presence of CAD and MI as well as the severity of CAD in Japanese patients undergoing CAG.

The Japanese population is well known to have a very low incidence and mortality of CAD [3], thought to be due to the Japanese diet, which is characterized by high intakes of soy products, fish, vegetables, rice, miso soup, seaweed, pickles, fruits, and green tea [1,2]. Notably, the Japanese diet is recognized to have some similarities to the Mediterranean diet [8], which is characterized by high intakes of whole grains, legumes, fish, vegetables, fruits, and olive oil [20,21] and is widely known to be associated with a reduced risk of CVD and CAD [20,21,22]. Among the components of the Japanese diet, the inclusion of fish, fruits, and vegetables is similar to the Mediterranean diet, and these foods are reported to have the strongest and most consistent protective associations with CVD and CAD [23,24,25]. The more distinctive foods and beverages of the Japanese diet are soy products and green tea. A meta-analysis of Japanese cohort studies reported that an increased green tea intake was associated with reduced CVD and HD/CAD combined mortality, whereas no significant association was found between the intake of soy products and CVD mortality [8]. However, this result cannot exclude the possibility of CVD mortality being reduced by the intake of soy products. While several foods in the Japanese diet were investigated for their association with CVD or CAD in cohort studies, the results were not always consistent [5]. Recently, increased attention has been paid to dietary patterns rather than individual nutrients or foods, because these foods are consumed in combination and correlate with one another and because it is difficult to examine their effects separately [7].

Several Japanese cohort studies investigated the association between the Japanese diet and CVD or CAD mortality [5,7,26]. However, the results were not always consistent, and most of those studies conducted their dietary surveys around 1990. The Japan Public Health Center (JPHC) cohort study conducted a dietary survey of 92,969 Japanese adults in 1990 [7]. That study investigated the Japanese diet, which consisted of eight foods (high intake of rice, miso soup, seaweeds, pickles, green/yellow vegetables, fish, and green tea; low intake of meat), and reported that adherence to the Japanese diet was associated with reduced mortality from CVD and HD, including CAD. Among the eight foods evaluated, the intakes of vegetables and green tea were also significantly associated with reduced CVD and HD mortality. The Japan Collaborative Cohort (JACC) study also conducted a dietary survey of 58,767 Japanese adults between 1988 and 1990 [26]. That study investigated the Japanese food score derived from seven foods (soy products, fish, vegetables, pickles, fungi, seaweed, and fruits). However, while a high Japanese food score was found to be associated with reduced CVD mortality in women, no such significant association was found in men. Furthermore, the Ohsaki National Health Insurance (NHI) cohort study evaluated dietary patterns in 40,547 Japanese adults in 1994 [5]. The Japanese dietary pattern was associated with reduced CVD mortality, but this study failed to show any significant association between the Japanese diet and CAD mortality. However, a recent meta-analysis of Japanese cohort studies by Shirota et al. [8] demonstrated that stronger adherence to the Japanese diet was associated with lower CVD and HD/CAD combined mortality rates. The present study investigated the association between the Japanese diet and the presence and severity of CAD in 802 Japanese patients undergoing CAG. The Japanese diet score, defined as the sum of the scores of intakes of fish, soy products, vegetables, seaweed, fruits, and green tea, was found to be low in patients with CAD and to be inversely associated with the presence of CAD and MI and the severity of CAD. Furthermore, the proportion of patients with a Japanese diet score of >6 was significantly lower in patients with CAD (36%) than in those without CAD (53%), indicating that the sensitivity and specificity of a Japanese diet score >6 for CAD (−) were 53% and 64%, respectively. Based on the results of our study, clinicians may thus recommend their patients at high risk for CAD to take a Japanese diet with a score of >6, which is equivalent to consuming more than all five foods ≥3 times/week and green tea ≥1 cups/day. Although the present study is a hospital-based, cross-sectional study, our results support the notion that the Japanese diet is protective against the development of CAD in the Japanese population.

In patients undergoing CAG, several studies previously reported the inverse associations between angiographically proven CAD and the intakes of fish [27], soy products, [28] and vegetables [29]. Regarding the association between CAD and the dietary patterns assessed when CAD was diagnosed in patients undergoing CAG, Waldeyer et al. [10] reported that a high Mediterranean diet score was inversely associated with the presence of severe CAD, defined as a SYNTAX score of ≥23, in 1121 patients undergoing CAG in Germany. In contrast, Oikonomou et al. [11] showed that the Western dietary pattern was positively associated with the presence of severe CAD in 188 patients with CAD in Greece. Furthermore, Kuhail et al. [30] also reported that an unhealthy dietary pattern was associated with severe CAD, defined as a Gensini score ≥20, in 423 patients with CAD in Iran. In Japan, we [15,16] and others [12,13] reported the inverse associations between green tea intake and the presence of CAD and MI in Japanese patients undergoing CAG. One case–control study by Sasazuki et al. [14] investigated the association between the intakes of fish, tofu, vegetables, and fruits and the presence of MI in 632 patients with MI and 1214 controls. They found that only tofu intake was significantly associated with MI in women but not in men. However, no study has reported the association between the Japanese diet and CAD or MI in patients undergoing CAG. We recently reported that green tea intake was inversely associated with the presence of CAD and MI in 612 Japanese patients undergoing CAG [15]. Interestingly, the inverse association between green tea intake and CAD and MI was found in patients with a high intake of vegetables or fruits but not in those with a low intake of vegetables or fruits, suggesting that green tea intake may have a synergistic effect on the protective role of vegetables and fruits against the development of CAD. The present study conducted in Japan demonstrated for the first time that the Japanese diet characterized by high intakes of fish, soy products, vegetables, seaweed, fruits, and green tea was inversely associated with the presence of CAD and MI in 802 patients undergoing CAG. We also showed that the Japanese diet score was independently and inversely associated with the presence of severe CAD, defined as 3-VD. We further found that the Japanese diet score was significantly, but weakly, inversely correlated with the numbers of >50% and >25% stenotic coronary segments (r = −0.12 and r = −0.14, respectively). Therefore, the results of these cross-sectional studies, including our own study, support the findings of cohort studies that healthy dietary patterns, such as the Japanese diet, play a protective role in the progression of CAD. Furthermore, since the intake of foods such as fish was reported to be associated with reduced progression of coronary artery stenosis [31], a further prospective study is warranted to look at the association between the retardation of stenosis progression and the Japanese diet.

Several limitations associated with the present study warrant mention. First, in our study, a semiquantitative FFQ was used to assess the intake of foods and beverages, as in many cohort studies, since the FFQ is the most common tool for investigating the association between diet and diseases [32]. FFQs have been shown to be fairly reproducible in studies of patients with CAG [14] as well as in cohort studies [33]. Using the FFQ, we previously reported the association between the intake of green tea and CAD or MI in patients undergoing CAG [15,16,17]. However, the reproducibility of our FFQ has not been evaluated. Furthermore, our FFQ did not ask about the individual portion size or the preparation of foods, including sodium use. The actual intake amounts of foods and nutrients as well as the sodium intake cannot be demonstrated by our FFQs. These are major limitations of our study. Second, because the dietary intake was assessed only at one time when CAG was performed, the study patients may have changed their dietary habits over time; however, changes in dietary habits were not considered. Third, our cross-sectional study patient population was much smaller than in most cohort studies. The sample size of our study (802 patients) was found to be enough to show a 0.41-fold lower risk of CAD in patients with the highest score (8–12) with a statistical power of 80% and an α value of 0.05, because 532 patients were estimated to be the adequate size with the proportion of CAD (64%). However, the small number of study patients is one of the major limitations. Fourth, as shown in Table 4, the final models in the multivariate analysis presented low Nagelkerke’s R^2^ values, which ranged from 0.15 to 0.27 for predicting CAD, 3-VD, and MI, indicating poor overall performance. These are one of the major limitations, and other dietary factors, such as sodium intake, and genetic factors may contribute to the development of CAD. Fifth, because our study was cross-sectional in nature, it cannot establish causality and only shows some associations. Finally, our study was performed in Japanese patients undergoing CAG, and they were considered to be a highly selected population who were at high risk for CAD. Our sampling method is considered to be convenience sampling. This may have caused some selection bias and may have confounded the results of our study. Furthermore, Scarano et al. [34] reported an inverse association between the Mediterranean diet and MI in European patients, but they found no such significant association in Chinese patients. Our results may therefore not be applicable to a general population or other ethnic populations.

## 5. Conclusions

The present study conducted in Japan demonstrated for the first time that the intakes of foods and green tea characteristic of the Japanese diet were low in patients with CAD and that the Japanese diet was inversely associated with the presence of CAD and MI as well as the severity of CAD in Japanese patients undergoing CAG. Although the present study is a hospital-based, cross-sectional study, our results support the notion that the Japanese diet plays a protective role against the development of CAD in the Japanese population.

## Figures and Tables

**Table 1:** Clinical characteristics of patients with and without CAD.

	CAD (−) (*n* = 291)	*p*-Value CAD (−) vs. CAD	CAD (*n* = 511)	MI (−) (*n* = 338)	MI (−) vs. MI (+)	MI (+) (*n* = 173)	CAD (−) vs. MI (+)
Age (years)	65 ± 12	<0.001	68 ± 11	70 ± 10	<0.001	65 ± 12	NS
Sex (male)	179 (62%)	<0.001	404 (79%)	267 (79%)	NS	137 (79%)	<0.05
BMI (kg/m^2^)	24.2 ± 10.6	NS	24.0 ± 3.8	24.2 ± 3.5	NS	23.7 ± 4.4	NS
Hypertension	181 (62%)	<0.001	392 (77%)	271 (80%)	<0.05	121 (70%)	NS
Systolic BP (mmHg)	131 ± 22	NS	133 ± 26	133 ± 23	NS	133 ± 31	NS
DM	42 (14%)	<0.001	173 (34%)	128 (38%)	<0.05	45 (26%)	<0.05
Smoking	115 (40%)	<0.001	279 (55%)	177 (52%)	NS	102 (59%)	<0.05
Hypercholesterolemia	116 (40%)	<0.001	293 (57%)	197 (58%)	NS	96 (55%)	<0.05
Statin	75 (26%)	<0.001	204 (40%)	146 (43%)	NS	58 (34%)	NS
LDL-cholesterol (mg/dL)	112 ± 29	0.026	117 ± 34	114 ± 33	0.009	123 ± 35	0.001
HDL-cholesterol (mg/dL)	58 ± 17	<0.001	49 ± 13	52 ± 13	<0.001	45 ± 11	<0.001

Data are shown as the mean ± SD or number (%) of patients. BMI: body mass index; BP: blood pressure; DM, diabetes mellitus; NS, not significant.

**Table 2:** Intakes of foods and beverages in patients with and without CAD.

	CAD (−) (*n* = 291)	*p*-Value CAD (−) vs. CAD	CAD (*n* = 511)	MI (−) (*n* = 338)	MI (−) vs. MI (+)	MI (+) (*n* = 173)	CAD (−) vs. MI (+)
Fish (times/week)							
<3	92 (32%)	<0.05	199 (39%)	123 (36%)	NS	76 (44%)	<0.05
3–4	159 (55%)		253 (50%)	178 (53%)		75 (43%)	
>4	40 (14%)	NS	59 (12%)	37 (11%)	NS	22 (13%)	NS
Soy products (times/week)							
<3	86 (30%)	<0.05	187 (37%)	122 (36%)	NS	65 (38%)	NS
3–4	124 (43%)		186 (36%)	120 (36%)		66 (38%)	
>4	81 (28%)	NS	138 (27%)	96 (28%)	NS	42 (24%)	NS
Vegetables (times/week)							
<3	41 (14%)	<0.05	118 (23%)	75 (22%)	NS	43 (25%)	<0.05
3–4	92 (32%)		172 (34%)	110 (33%)		62 (36%)	
>4	158 (54%)	<0.05	221 (43%)	153 (45%)	NS	68 (39%)	<0.05
Seaweed (times/week)							
<3	144 (49%)	NS	288 (56%)	185 (55%)	NS	103 (60%)	NS
3–4	97 (33%)		161 (32%)	109 (32%)		52 (30%)	
>4	50 (17%)	<0.05	62 (12%)	44 (13%)	NS	18 (10%)	NS
Fruits (times/week)							
<3	65 (22%)	<0.05	159 (31%)	103 (30%)	NS	56 (32%)	NS
3–4	75 (26%)		134 (26%)	86 (25%)		48 (28%)	
>4	151 (52%)	<0.05	218 (43%)	149 (44%)	NS	69 (40%)	<0.05
Meat (times/week)							
<3	130 (45%)	NS	256 (50%)	170 (50%)	NS	86 (50%)	NS
3–4	131 (45%)		208 (41%)	140 (41%)		68 (39%)	
>4	30 (10%)	NS	47 (9%)	28 (8%)	NS	19 (11%)	NS
Green tea (cups/day)							
<1	51 (18%)	<0.05	129 (25%)	84 (25%)	NS	45 (26%)	NS
1–3	159 (55%)		283 (55%)	183 (54%)		100 (58%)	
>3	81 (28%)	<0.05	99 (19%)	71 (21%)	NS	28 (16%)	<0.05
Coffee (cups/day)							
<1	106 (36%)	NS	154 (30%)	106 (31%)	NS	48 (28%)	NS
1–3	161 (55%)		309 (60%)	207 (61%)		102 (59%)	
>3	24 (8%)	NS	48 (9%)	25 (7%)	NS	23 (13%)	NS
Alcohol (times/week)							
<1	127 (44%)	<0.05	273 (53%)	172 (51%)	NS	101 (58%)	<0.05
≥1	78 (27%)		129 (25%)	89 (26%)		40 (23%)	
Every day	86 (30%)	<0.05	109 (21%)	77 (23%)	NS	32 (18%)	<0.05
**Japanese diet score**	6.3 ± 2.7	<0.001	5.4 ± 2.9	5.6 ± 2.9	NS	5.2 ± 2.8	<0.001
**Score > 6**	153 (53%)	<0.001	185 (36%)	129 (38%)	NS	56 (32%)	<0.05

**Table 3:** The proportion of CAD, 3-VD, and MI in the three groups of Japanese diet score.

Japanese Diet Score	T1 Group 0–4 (*n* = 271)	T2 Group 5–7 (*n* = 295)	T3 Group 8–12 (*n* = 236)	*p*-Value T1 vs. T3
Age (years)	62 ± 11	67 ± 11	72 ± 10	<0.001
Sex (male)	230 (85%)	211 (72%)	142 (60%)	<0.05
BMI (kg/m^2^)	25.4 ± 10.8	23.8 ± 3.7	23.0 ± 4.1	0.001
Hypertension	190 (70%)	220 (75%)	163 (69%)	NS
Systolic BP (mmHg)	132 ± 26	132 ± 24	133 ± 22	NS
DM	70 (26%)	78 (26%)	67 (28%)	NS
Smoking	175 (65%)	134 (45%)	85 (36%)	<0.05
Hypercholesterolemia	134 (49%)	155 (53%)	120 (51%)	NS
Statin	85 (31%)	111 (38%)	83 (35%)	NS
LDL-cholesterol (mg/dL)	118 ± 32	114 ± 33	113 ± 33	NS
HDL-cholesterol (mg/dL)	50 ± 13	53 ± 15	55 ± 16	0.002
CAD	196 (72%)	185 (63%)	130 (55%)	<0.05
Number of >50% stenotic segments	2.0 [0.0, 3.5]	1.0 [0.0, 3.0]	1.0 [0.0, 3.0]	0.002
Number of >25% stenotic segments	3.0 [1.0, 5.0]	2.0 [0.0, 4.0]	1.0 [0.0, 4.0]	<0.001
3-VD	59 (22%)	51 (17%)	38 (16%)	NS
MI	69 (25%)	70 (24%)	35 (15%)	<0.05

Data are shown as the mean ± SD or number (%) of patients, except for the numbers of >50% and >25% stenotic segments, which are expressed as the median and interquartile range. BMI: body mass index; BP: blood pressure; DM, diabetes mellitus; CAD, coronary artery disease; 3-VD, three-vessel disease; MI, myocardial infarction; NS, not significant.

**Table 4:** Adjusted odds ratios (95% CI) for the proportion of CAD, 3-VD, and MI by the tertiles of Japanese diet score.

Japanese Diet Score	T1 Group 0–4 (*n* = 271)	T2 Group 5–7 (*n* = 295)	T3 Group 8–12 (*n* = 236)	Nagelkerke R^2^
** CAD**				
Adjusted odds ratio (Model 1)	1	0.59 (0.40–0.85) **	0.39 (0.26–0.59) *	0.110
Adjusted odds ratio (Model 2)	1	0.60 (0.40–0.90) ^#^	0.42 (0.27–0.65) *	0.258
Adjusted odds ratio (Model 3)	1	0.60 (0.40–0.90) ^#^	0.41 (0.26–0.63) *	0.274
** 3-VD**				
Adjusted odds ratio (Model 1)	1	0.59 (0.38–0.92) ^#^	0.46 (0.28–0.76) **	0.077
Adjusted odds ratio (Model 2)	1	0.57 (0.37–0.90) ^#^	0.45 (0.27–0.74) **	0.140
Adjusted odds ratio (Model 3)	1	0.57 (0.37–0.90) ^#^	0.45 (0.27–0.74) **	0.147
** MI**				
Adjusted odds ratio (Model 1)	1	0.98 (0.66–1.45)	0.60 (0.37–0.96) ^#^	0.033
Adjusted odds ratio (Model 2)	1	1.08 (0.72–1.63)	0.64 (0.40–1.04)	0.171
Adjusted odds ratio (Model 3)	1	1.06 (0.70–1.61)	0.61 (0.38–0.99) ^#^	0.180

The dependent variables were the presence of CAD, 3-VD, or MI. Model 1 was adjusted for age and sex; Model 2 was adjusted for age and sex, hypertension, hypercholesterolemia, HDL-cholesterol levels, statin use, DM, and smoking; Model 3 was further adjusted for the intake of meat, coffee, and alcohol in addition to the same covariates in Model 2.; * *p* < 0.001, ** *p* < 0.01, ^#^ *p* < 0.05. CAD, coronary artery disease; 3-VD, three-vessel disease; MI, myocardial infarction.

## Data Availability

The data that support the findings of this study are available from the corresponding author on reasonable request.
